# Simultaneous identification of multi-combustion-intermediates of alkanol-air flames by femtosecond filament excitation for combustion sensing

**DOI:** 10.1038/srep27340

**Published:** 2016-06-02

**Authors:** Helong Li, Wei Chu, Huailiang Xu, Ya Cheng, See-Leang Chin, Kaoru Yamanouchi, Hong-Bo Sun

**Affiliations:** 1 State Key Laboratory on Integrated Optoelectronics, College of Electronic Science and Engineering, Jilin University, Changchun 130012, China; 2 State Key Laboratory of High Field Laser Physics, Shanghai Institute of Optics and Fine Mechanics, Chinese Academy of Sciences, Shanghai 201800, China; 3 Department of Physics and Center for Optics, Photonics and Laser, Laval University, Québec, G1V 0A6, Canada; 4 Department of Chemistry, School of Science, The University of Tokyo, 7-3-1 Hongo, Bunkyo-ku, Tokyo, 113-0033, Japan; 5 College of Physics, Jilin University, Changchun 130012, China

## Abstract

Laser filamentation produced by the propagation of intense laser pulses in flames is opening up new possibility in application to combustion diagnostics that can provide useful information on understanding combustion processes, enhancing combustion efficiency and reducing pollutant products. Here we present simultaneous identification of multiple combustion intermediates by femtosecond filament excitation for five alkanol-air flames fueled by methanol, ethanol, n-propanol, n-butanol, and n-pentanol. We experimentally demonstrate that the intensities of filament-induced photoemission signals from the combustion intermediates C, C_2_, CH, CN increase with the increasing number of carbons in the fuel molecules, and the signal ratios between the intermediates (CH/C, CH/C_2_, CN/C, CH/C_2_, CN/CH) are different for different alkanol combustion flames. Our observation provides a way for sensing multiple combustion components by femtosecond filament excitation in various combustion conditions that strongly depend on the fuel species.

Diagnostics of combustion species, temperature and reaction process is of significance in both fundamental science and engineering application for enhancing combustion efficiency and reducing pollutant products^1^,^2^. A variety of measurement techniques have been developed for sensing combustion intermediates, which can provide detailed information on analyzing combustion species formation and consumption. In particular, laser-based spectroscopic techniques such as laser-induced fluorescence (LIF), coherent anti-Stokes Raman scattering, and polarization spectroscopy have shown the capabilities of noninvasive, sensitive, and high-speed measurements with high spatial resolutions^3^,^4^. For example, by using planar LIF technique, measurements of a large number of intermediate species in combustion, such as OH, CH, and CO have been demonstrated with high detection sensitivity.

Recently, a technique called filament-induced nonlinear spectroscopy (FINS) was developed for sensing atmospheric constituents^5^,^6^. Unlike the LIF technique that requires the laser wavelength to be resonant with the species under study^7^,^8^,^9^,^10^, the FINS technique is based on the unique nonlinear optical phenomenon of femtosecond laser filamentation, resulting from the dynamic equilibrium between Kerr self-focusing and the defocusing effect of the self-generated low-density plasma when intense femtosecond laser pulses propagates in optical media^11^,^12^,^13^. The laser intensity inside a filament is high enough to induce multiphoton excitation of atmospheric species resulting in characteristic fingerprint fluorescence^6^. More recently, it was shown that using the FINS technique, fingerprint fluorescence emissions from multiple combustion intermediates such as CH, C_2_ and CN radicals and atomic C and H in an ethanol-air flame could be simultaneously probed, which provides the possibility of simultaneous monitoring of multiple combustion intermediate species^14^. Since then, based on the FINS technique, new phenomena and new effects in the flame filament have been explored. For example, by comparing FINS spectrum with those obtained from ns-LIBS and the combustion emission itself in the ethanol-air flame, it was demonstrated that the fingerprint fluorescence produced in a flame filament mainly come from the excitation of intermediate species existing in the combustion flame, but not from the fragments generated by the dissociation of parent molecules by the intense femtosecond laser field^15^. It was also discovered that fingerprint emission from the specific species of CN in an ethanol-air flame array can be amplified through amplified spontaneous emission (ASE) by observing CN fluorescence in a backward direction of the laser propagation as a function of the plasma length^16^. This lasing action was suggested to be a potential method that can overcome fluorescence quenching effect and improve the signal-to-noise ratio especially for the high-temperature and high-pressure engine combustion environments. In particular, it was revealed recently that the critical power and clamping intensity in flames are much smaller than those in air^17^, which provides new insights into the understanding of interaction of combustion flames with femtosecond laser filamentation.

However, up to now all the investigations on combustion in the flame filament have been focused on the ethanol-air flame. Since in the combustion, the fuel undergoes complex decomposition process, produces plenty of rich radicals and induces a large number of combined reactions, reliable analysis of combustion processes becomes more difficult when the fuel molecules become larger^18^,^19^. In addition, the interaction processes of laser filamentation with combustion flames of different fuels may also be different. For example, the analysis of the LIBS spectrum confirms that the laser breakdown threshold in flames with fuel molecule consisting of more carbon atoms is noticeably different from that in flames with fuel molecule consisting of less carbon atoms^20^. In particular, femtosecond laser filamentation is a highly nonlinear process, and thus its properties such as the clamped laser intensity are strongly dependent on the working environments, that is, the fuels used in the combustion conditions in our current study. Therefore, the investigation regarding the fuel effect on the functionality of FINS in combustion diagnostics is anticipated. In the present study, we systematically investigate a series of fuel-air flames (i.e., methanol, ethanol, n-propanol, n-butanol, and n-pentanol) using the FINS technique. Analysis of the FINS spectra demonstrates that the fluorescence signals of the intermediates and their ratios depends strongly on the number of carbon atoms in the fuel molecule at different fuel-air flames. Even so, our results demonstrate that the FINS technique can be used for sensing combustion intermediates of different combustion conditions, which is of significance for rationalizing the combustion reaction dynamics, and shed more light on the understanding of the multi-component combustion diagnostics.

## Results and Discussion

### Fingerprint emissions induced by filamentation from the n-pentanol-air flame

**Figure 1:**
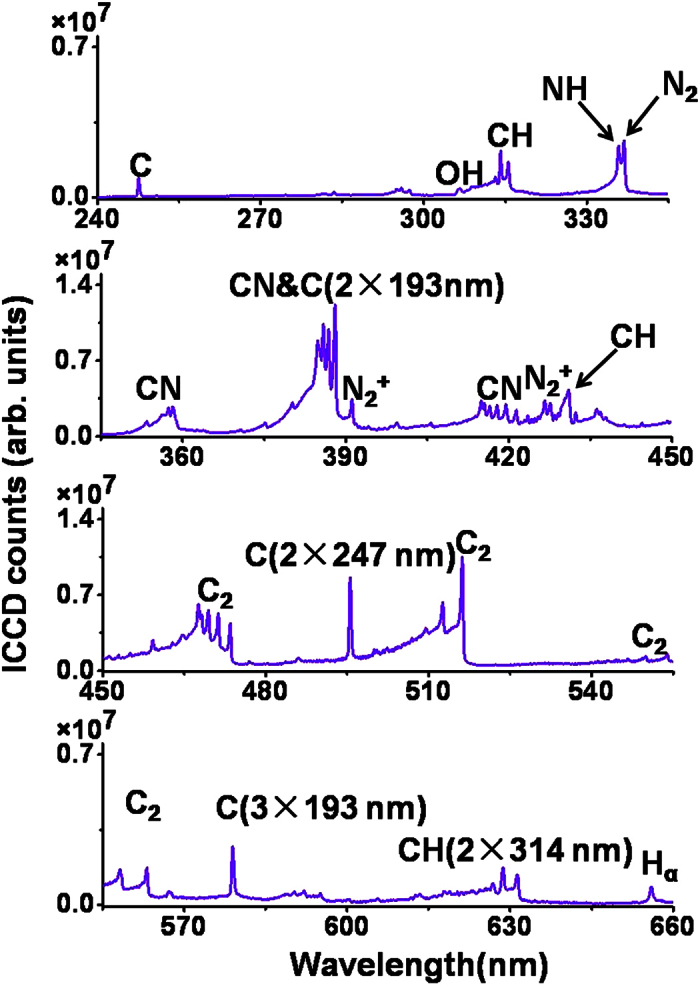
Filament-induced nonlinear spectrum of the n-pentanol-air flame on an alcohol burner in the range of 240–660 nm.

Figure 1 shows a filament-induced spectrum of the n-pentanol (C_5_H_12_O)-air flame in the spectral range of 240–660 nm. The measurement was performed with the filament formed at a distance of 17 mm above the burner wick. The ICCD gate width and delay were set to Δ*t* = 210 ns and *t* = −5 ns, respectively (note that the laser pulse arriving time at the interaction zone is t = 0 ns). All the results shown in this work were accumulated over 300 laser shots (for experimental details, see Methods). In addition, the measurements of the filament-induced spectra show good repetitiveness due to the laminar nature of the alcohol-air combustion flame. As shown in Fig. 1, the spectral bands can be assigned to the combustion intermediates of CN, CH, OH, NH, C and H. The spectral bands at 337 nm resulting from the nitrogen molecule N_2_ in air can also be observed^8^. The main spectral feature shown in Fig. 1 is similar to that in the FINS spectrum of the ethanol-air flame obtained previously in ref. [14] although n-pentanol molecule (CH_3_(CH_2_)_4_OH) is much larger than ethanol molecule (CH_3_CH_2_OH). This observation clearly indicates that the combustion reactions of the two different fuels produce identical combustion intermediate species, which are determined by the compositions (C, H and O) of the two fuels.

**Figure 2:**
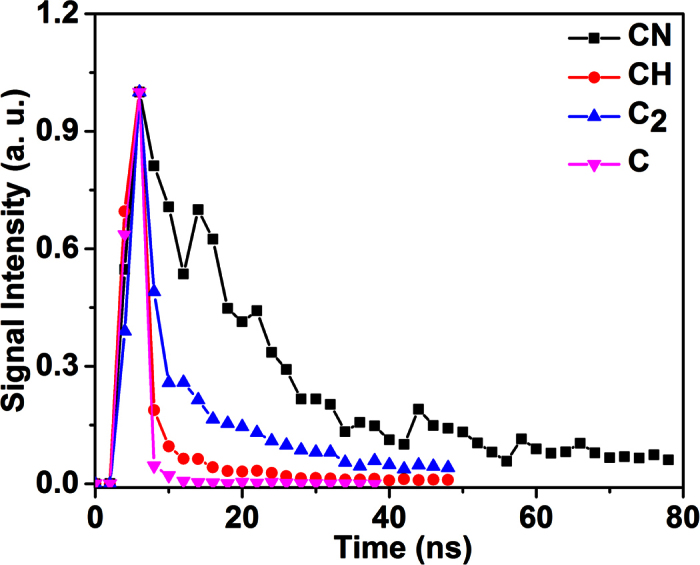
The decay curves of filament-induced fluorescence from the n-pentanol (C_5_H_12_O)-air flame. In the measurements, both of the ICCD gate width and the delay step were set to 2 ns.

It should be pointed out that the FINS signals recorded with the employed ICCD gate width and time delay integrated all the fluorescence in the time domain because the decay times of the combustion intermediates are typically in the range of 10–30 ns, as shown in Fig. 2. On the other hand, it can be seen from Fig. 2 that the decays are different for different combustion intermediates. Therefore, if the ICCD gate width was set too small to collect all the fluorescence in the time domain, the measured FINS signals from different combustion intermediates could be varied differently. It should also be emphasized that the fluorescence signals obtained in the FINS technique could not be directly used, similarly to LIF or LIBS^2^, to evaluate the concentrations of combustion intermediates due to the calibration difficulty in complex combustion environments, but they can be used to show qualitatively the relative concentration distribution of the combustion intermediates in flames.

In addition, we performed a measurement of the FINS signals with different pulse durations of the pump laser. It was found that the shorter the pulse duration becomes, the stronger the FINS signal becomes (not shown). Since the clamping intensity inside a gas filament becomes lower when the pulse duration increases^13^, the stronger signals with shorter pulse duration can be ascribed to the higher clamping intensity inside the filament. However, it should be pointed out that for a pulse with a given set of laser parameters, the effect of intensity clamping inside filaments would maintain a homogeneous interaction zone for the reaction. That is to say, the FINS signals from the same chemical species are produced by the interaction with essentially the same intensity.

### FINS spectra produced from different alkanol-air flames

**Figure 3:**
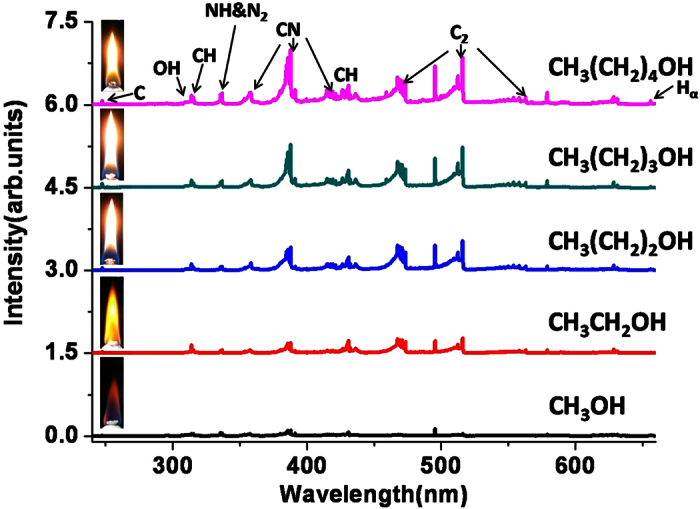
Filament-induced nonlinear spectra for five flame conditions with the filament at 17 mm above the burner wick.

Shown in Fig. 3 are the FINS spectra measured from the combustion flames of five alkanol fuels, i.e., methanol (CH_3_OH), ethanol (CH_3_CH_2_OH), n-propanol (CH_3_(CH_2_)_2_OH), n-butanol (CH_3_(CH_2_)_3_OH), and n-pentanol (CH_3_(CH_2_)_4_OH). In this measurement, all the FINS spectra were carried out with the filament formed at a distance of 17 mm above the burner wick. The insets in Fig. 3 are the combustion flame photos of the five fuels taken by a digital camera. It can be seen from Fig. 3 that all the FINS spectra of the five alkanol-air flames show identical spectral bands and atomic lines, but the spectral intensities of intermediate species are noticeably different for different fuels. The identical combustion intermediate species show the nature of the five fuels composed with the same atomic species of C, H and O; meanwhile the different spectral intensities show the capability of the FINS technique in distinguishing quantitatively the combustion intermediates of different fuels.

**Figure 4:**
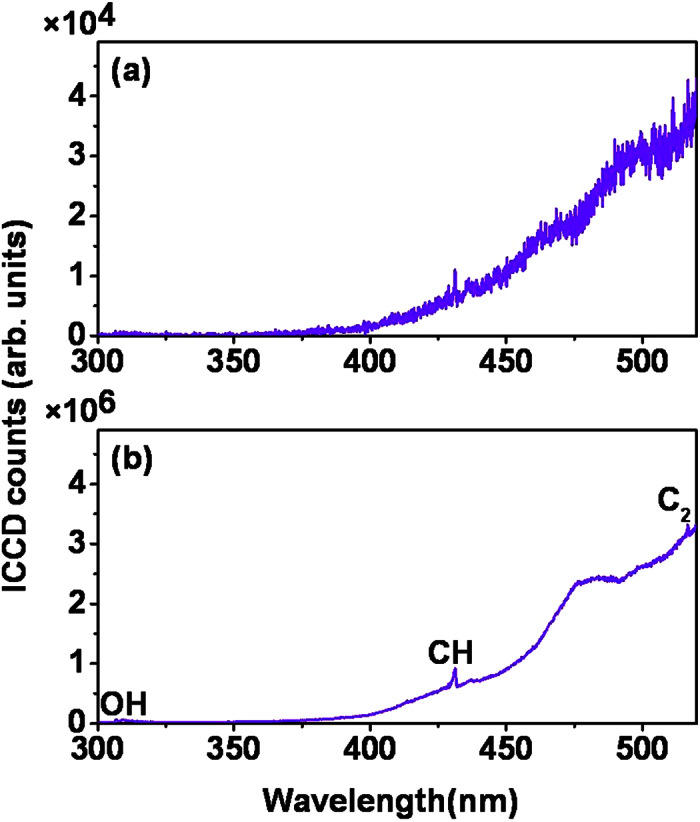
The emission spectra of the n-pentanol-air flame itself with the laser off for the ICCD gate width of (a) 210 ns and (b) 21 μs.

The FINS signals appear to become stronger when the carbon-carbon bond chain of the molecule becomes longer. It should be pointed out that the FINS signals from all the alkanol-air flames are much stronger than the emissions from the flames themselves with the laser off. As shown in Fig. 4a, when all the experimental conditions were kept the same as those in the FINS measurement except for blocking the laser, the measured signals of free radicals from n-pentanol-air flame itself are too weak to be observed. When the ICCD gate width was increased from 210 ns to 21 μs, the measured emissions (Fig. 4b) from the free radicals of OH, CH and C_2_ can be seen. However, the signals are still about one order of magnitude smaller than that shown in Fig. 1, and the continuum emission from the flame is dominant in the spectrum. This indicates that the signal from the n-pentanol-air flame itself is about three orders of magnitude weaker than that obtained from the FINS measurement. The much weaker signals from the flames themselves show that the population in the electronically excited states of the combustion intermediates in the flame determined by the Boltzmann distribution at typical temperature of 700–1000 K^17^ in the alkanol-air flames is many orders of magnitude smaller than their population in the electronic ground states.

### Dependence of FINS signals on the number of carbon atoms in fuel molecules

**Figure 5:**
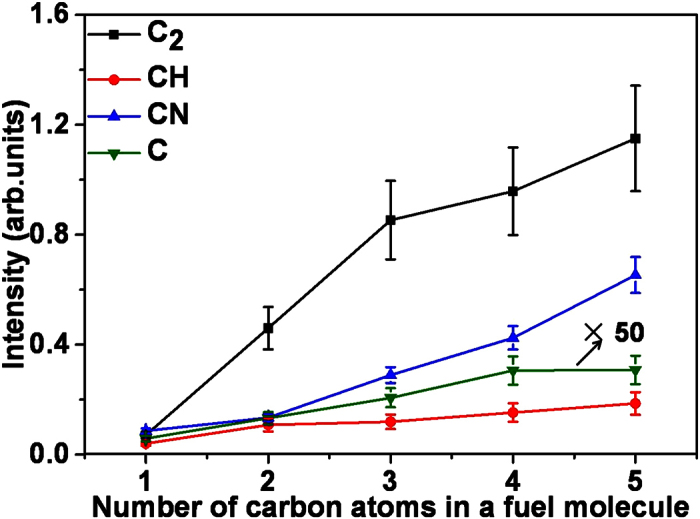
Signal intensities of C_2_, CN, C, and CH, obtained at 17 mm for the series of C1–C5 alkanol flames.

To see closely the variation of FINS signal intensities, we plot the dependence of the photoemission signal intensities of the four intermediate species, C, CH, CN and C_2_ on the five fuel molecules in Fig. 5. For simplicity, the number of the carbon atoms in the five fuel molecules is used to represent the corresponding molecule (i.e, C1: methanol, C2: ethanol, C3: n-propanol, C4: n-butanol, and C5: n-pentanol). The signal intensities of the four species in Fig. 5 are obtained by integrating the spectral line of atomic C in the range of 247.3–248.1 nm, the two bands of CH radical in the ranges of 314.0–315.8 nm and 428.1–431.7 nm, the three bands of CN radical in the ranges of 354.1–359.0 nm, 386.3–388.6 nm and 414.1–421.8 nm, and the three bands of C_2_ radical in the ranges of 465.4–475.1 nm, 510.2–516.9 nm, and 552.0–563.5 nm, respectively. The signal uncertainties are about 10–25%, which mainly result from the swing of the flame.

It can be clearly seen in Fig. 5 that for all the four species, the signal intensities increase as the number of the carbon atom(s) in the fuel molecules becomes larger. Such increased fluorescence signals for fuels with more carbon atoms in FINS are consistent with previous LIF and LIBS measurements. For examples, Sutton *et al*. showed low-pressure LIF signals from flames for C1–C4 alkane fuels^21^, and found that the signal intensity of the combustion intermediates increases as the fuel changes from methane (C1), ethane (C2), propane (C3) to butane (C4). Michalakou *et al*. measured the H/O and C/O ratios using LIBS technique for three types of fuels (CH_4,_ C_2_H_4_, and C_3_H_8_), and demonstrated that the H/O and C/O ratios are strongly dependent to the fuel species^22^. The formation of the combustion intermediate species (C, CH, C_2_) in the flames were suggested to mainly come from decomposition of fuel molecules, in which the final decomposition steps for hydrocarbons with higher carbon number are similar to that of the primary decomposition pathway of those with lower carbon number^20^. This leads to the result that the concentration of intermediate species produced from the flames with fuel molecule consisting of more carbon atoms is much higher.

On the other hand, chemical reactions of a variety of intermediates and molecules during combustion in the flames could produce the intermediate of CN (for example through C_2_ + N_2_ = 2CN)^23^ giving rise to the higher concentration of CN for larger fuel molecules. Furthermore, it can be seen from Fig. 5 that the slope for C_2_ is much steeper than those for other intermediates as the number of carbon atom(s) in the fuel molecules changes from 1 to 3. For methanol (CH_3_OH), it can be undoubtedly concluded that the generation of C_2_ is not due to decomposition of parent molecule since the carbon-carbon bond is absent in the parent molecule. Therefore, when the number of carbon atoms in the fuel molecules becomes larger, the decomposition of the molecules might make additional contribution to the product of C_2_, leading to the steeper slope. It is worth stressing that the CN and C_2_ signal intensities in the n-pentanol-air flame are about one order of magnitude stronger than those in the methanol-air flame.

### Identification of the C1-C5 flames

**Figure 6:**
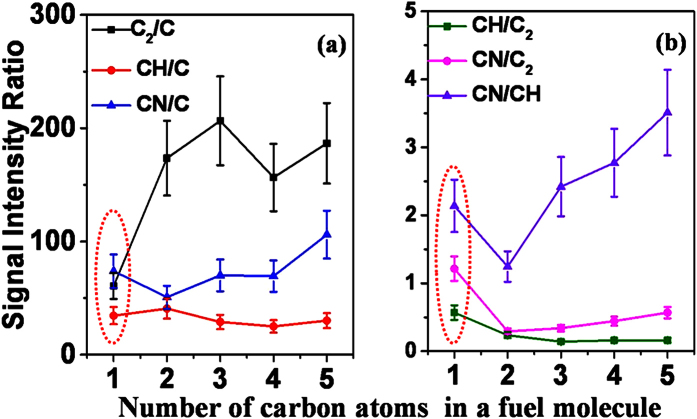
Signal ratios of intermediate species for five flame conditions.

Since the signal intensities of the intermediates in Fig. 5 increase at different slopes, we thus check the possibility to discriminate different fuels by comparing the fluorescence intensity ratios of different combustion intermediates in the FINS spectra, which is a common method to demonstrate the differences of various fuel-air flames^22^. As a result, the calculated ratios of the signal intensities between different intermediate species are shown in Fig. 6. For clarity, we plot respectively the ratios of C_2_/C, CN/C, CH/C in Fig. 6a and those of CH/C_2_, CN/C_2_, CN/CH in Fig. 6b as a function of the number of the carbon(s) since the C signal is much weaker than other intermediates. It can be clearly seen from Fig. 5 that except for the methanol-air flame (dashed circles), the ratio values for other four fuel-air flames show certain dependences on the number of the carbon atoms in the fuel molecules. That is, the ratios of CN/C, CN/CH, CN/C_2_ increase, but those of CH/C and CH/C_2_ decrease as the number of the carbon atoms of fuels increases. Therefore, based on the difference in these ratio values, the four fuel-air flames can be easily distinguished. In addition, the ratios of C_2_/C show a large fluctuation when the fuels change from ethanol to n-pentanol, but they are much larger than that in the methanol-air flame, which can be used to discriminate the methanol-air flame from other four fuel-air flames. The ratio difference between the methanol-air flame and other fuel-flames may result from the fact that the formation of C_2_ molecule in the methanol-air flame is obviously different from other flame conditions.

## Summary

We have systematically investigated the effect of fuels on the feasibility of FINS for sensing multiple intermediates of combustion flames with five types of fuels including methanol, ethanol, n-propanol, n-butanol, and n-pentanol. Comparison in the FINS spectra of different fuel-air flames demonstrates that the fluorescence intensities of the intermediates strongly depend on the number of carbons at different fuel-air flames. The fluorescence signals of all the four intermediates of C, C_2_, CH, CN increase as the number of carbons in the fuel molecules increases, but they show different slopes. The latter provides a way for the discrimination of the C1–C5 alkanol-air flames by comparing the differences of the signal ratios of the intermediates in different flames. Since the availability of high-power femtosecond laser system with high repetition rate of up to 10 kHz, our results reveal the possibility for high-speed monitoring of multiple combustion intermediates by means of femtosecond laser filament excitation.

## Methods

The experiments were carried out with a 0.6 mJ/100 fs, 1 kHz Ti:sapphire laser system. The laser pulses were focused by a fused-silica lens of 200 mm focal length into the fuel-air flames on an alcohol burner to generate a single filament with the length of ∼1 cm. The burner was fixed on an X-Y-Z translation stage, which could control the interaction positions between femtosecond laser filament and the flames. The flames were surrounded by a top-open black box to avoid the wind from the laboratory air conditioner that may cause a strong swing of the flame. Characteristic fingerprint emissions from the flame filament were collected using a fused-silica lens (50.8 mm in diameter, 60 mm focal length) from the side of the laser propagation direction and then focused on the entrance slit of a spectrometer (Andor Shamrock SR-303i) coupled with a gated intensified charge coupled device (ICCD, Andor iStar) in a 2f-2f imaging scheme. The entrance slit width for the spectrometer was set to 100 μm. The fluorescence was dispersed by a grating of 1200 grooves/mm (blazed wavelength at 500 nm) and detected by the ICCD camera.

## Additional Information

**How to cite this article**: Li, H. *et al*. Simultaneous identification of multi-combustion-intermediates of alkanol-air flames by femtosecond filament excitation for combustion sensing. *Sci. Rep.* **6**, 27340; doi: 10.1038/srep27340 (2016).
